# Genetic population structure and demographic history of the endemic fish *Paralichthys olivaceus* of the Northwest Pacific Ocean

**DOI:** 10.1002/ece3.9506

**Published:** 2022-11-15

**Authors:** Cheng‐He Sun, Fan Yang, Qi Huang, Xiao‐Shu Zeng, Ya‐Nan Zhang, Sha Li, Jian‐Feng Yu, Qun Zhang

**Affiliations:** ^1^ Department of Ecology and Institute of Hydrobiology Jinan University Guangzhou China; ^2^ Chinese Sturgeon Research Institute China Three Gorges Corporation Yichang Hubei China; ^3^ Hubei Key Laboratory of Three Gorges Project for Conservation of Fishes Yichang Hubei China

**Keywords:** Genetic diversity, Japanese flounder, Ocean current, Paralichthyidae, Phylogeography

## Abstract

The Northwest Pacific marginal waters comprising the South China Sea, East China Sea, Yellow Sea, and the Sea of Japan have unique geomorphic features. The Japanese flounder *Paralichthys olivaceus*, which is endemic to the Northwest Pacific, has high nutritional, economic, and ecological value. To allow the examination of the demographic history and population structure of the most common *P. olivaceus* species range over the five marginal seas (East China Sea, Yellow Sea, Bohai Sea, Northwest Pacific Ocean, and the Sea of Japan), the mitochondrial DNA control region of 91 individuals from six populations in China was sequenced. These sequences were combined with 233 sequences from four populations distributed in the Northwestern Pacific Ocean for analysis. Higher levels of nucleotide diversity (0.032 ± 0.016) and haplotype diversity (0.996 ± 0.001) were observed. The peripheral Fuqing population in the East China Sea had the relatively lowest genetic diversity and highest differentiation. Furthermore, when the results of the isolation by distance test, spatial analysis of molecular variation and geographic barrier analysis are also considered, there is a clear need to prioritize resource conservation and enhancement measures in this area. The phylogenetic trees, structure assignment test, and haplotypes network revealed no significant differences in the genealogical structure among ten populations. Mismatch distribution analysis, Bayesian skyline plots, and neutrality tests suggested that *P. olivaceus* experienced population expansion during the Pleistocene. Ocean currents and climate change play important roles in shaping the geographical distribution and genetic population structure of *P. olivaceus*.

## Graphical abstract

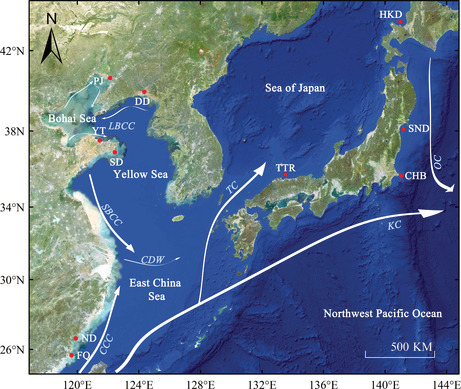

## 1 INTRODUCTION

Marginal water ecosystems in the Northwest Pacific are exposed to intense anthropogenic stresses, such as pollution and overfishing (Halpern et al., 2008; Ni et al., 2014; Yamashita et al., 2021; Zhang et al., 2020), and are affected by the complex ocean current patterns in the region. During the Last Glacial Maximum, the sea level in the Northwest Pacific decreased by 120–140 m. Marginal seas in the region were separated by land, forming many independent small sea areas with fragmented habitats (Lambeck et al., 2002; Voris, 2000; Zhao et al., 2022). The processes of sea‐level rise and fall have greatly impacted the species formation and genetic structure of organisms in the marginal waters of the Northwest Pacific (Liu et al., 2007; Ni et al., 2014). Many researchers have used molecular biology methods to evaluate the effects of regression, sea immersion, and sea habitat fragmentation on organisms in this region (Bae et al., 2020; Tang et al., 2010). The dispersal ability in the early life cycle of marine organisms can determine the genetic structure of marine biological populations to some extent (Caley et al., 1996). Through the action of ocean currents, groups of marine organisms with long pelagic larval durations may have genetic connectivity with others in different geographic locations. This connectivity may contribute to the lack of obvious genetic structures among many marine organisms with large geographical ranges (Blanco‐Bercial & Bucklin, 2016; Grant & Bowen, 1998; Hewitt, 2000).

The Japanese flounder *Paralichthys olivaceus* (Temminck & Schlegel, 1846) belongs to the order Pleuronectiformes and the family Paralichthyidae. *Paralichthys olivaceus* lives in bottom waters with warm temperatures and has important economic and ecological value (Hamidoghli et al., 2020; Kim et al., 2010; Sekino, 2006; Shigenobu et al., 2013). The Japanese flounder is the only member of *Paralichthys* along the coast of Asia, where it mainly feeds on crustaceans and small fish. It is widely distributed in the coastal areas of China, the Korean Peninsula, and Japan (Fujii & Nishida, 1997; Sekino et al., 2002; Zhang et al., 2019). The number of eggs laid by a 480 mm sexually mature female *P. olivaceus* is approximately 2 × 10^5^, whereas 600 mm sexually mature females lay approximately 4 × 10^5^ (Ochiai & Tanaka, 1986). The eggs and larvae can float on ocean waves for 25–50 days (Ochiai & Tanaka, 1986; Xu et al., 2012); the long floating period of the fish larvae and juveniles enables *P. olivaceus* to diffuse over a long distance with the ocean current. In theory, this diffusion strengthens connectivity between various geographical groups. Some of the characteristics of *P. olivaceus*, such as its large spawning numbers, large eggs, long floating period of larvae, and spawning migration (Kim et al., 2010; Sekino et al., 2002), are suitable for studying the impact of recontact of marine fish on the genetic structure of the population after habitat fragmentation in the Quaternary ice age.

Many studies of the mitochondrial DNA (mtDNA) of *P. olivaceus* have been conducted in recent years (Ando et al., 2016; Sekino et al., 2002; Yamashita et al., 2021) to clarify the genetic population structure of this fish. A study of the populations of *P. olivaceus* in the Sea of Japan (Fujii & Nishida, 1997) revealed high variability in the mtDNA control region. Analysis of the sequences of the mtDNA ND2 and ND5 genes indicated the presence of separate stocks among the northern and southern waters of the Pacific coast of Tohoku, Japan (Shigenobu et al., 2013). The highest rates of base substitutions and insertion/deletion events in mtDNA have been detected in the first half of the control region (adjacent to the proline transfer RNA gene) (Fujii & Nishida, 1997; Saccone et al., 1987). Therefore, in this study, we selected the first half of the mtDNA control region as the amplification region. The most common *P. olivaceus* species range was used as the sampling area, as dense sampling across species ranges is necessary to understand phylogeographic dynamics. We analyzed the population structure and demographic history to describe the geographic distribution patterns of *P. olivaceus* in all marginal seas of the Northwestern Pacific.

## 2 MATERIALS AND METHODS

### 2.1 Sample collection and DNA isolation

**FIGURE 1:**
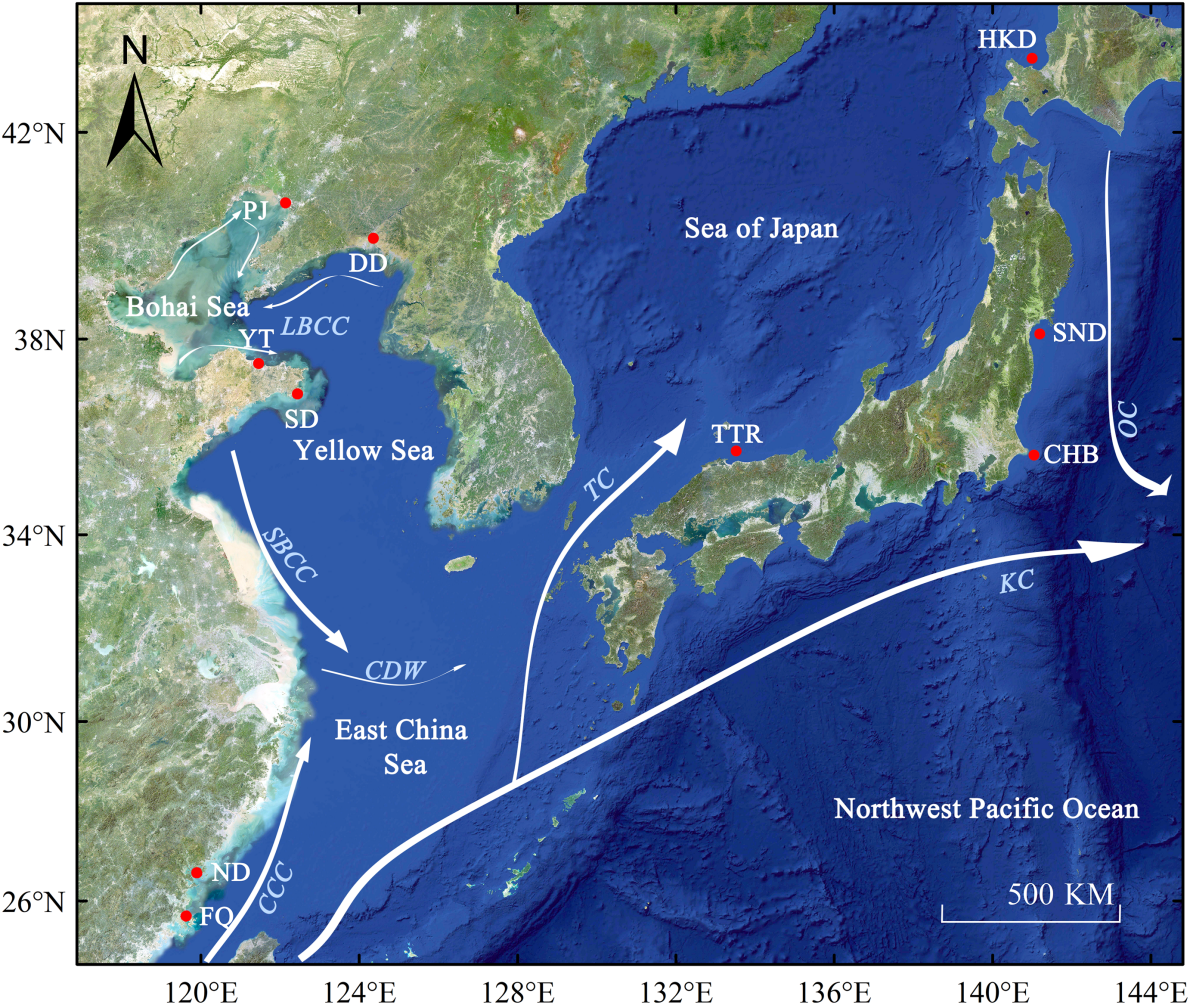
Map of China sea coastal waters showing sampling locations and flow directions of coastal currents and ocean currents (from: Ni et al., 2014). Geographic location of sampling locations of the study area are shown in Table 1. Abbreviations: CCC, China Coastal Currents; CDW, Changjiang diluted water; KC, Kuroshio Currents; LBCC, Lubei Coastal Current; OC, Oyashio Currents; SBCC, Subei Coastal Current; TC, Tsushima Currents.

Ninety‐one *P. olivaceus* specimens were collected from six geographical locations across Chinese coastal waters from 2004 to 2008 (Figure 1; Table 1). All individuals were identified based on their morphological characteristics (Nakabō, 2002; Nakabo & Doiuchi, 2013). A piece of muscle tissue was collected from each individual and stored at −20°C. Genomic DNA was extracted using standard phenol–chloroform extraction protocols with proteinase K treatment (Sambrook et al., 1989).

**TABLE 1:** Sampling information for *Paralichthys olivaceus*

Marginal Sea	Location	Coordinates	Collection dates	Sample size	Codes	Reference
Sea of Japan	Hokkaido Prefecture	141° E, 44° N	2000	50	HKD	Sekino et al. (2002)
	Tottori Prefecture	134° E, 36° N	1998	69	TTR	
Northwest Pacific Ocean	Chiba Prefecture	141° E, 36° N	1999	71	CHB	
	Sendai Bay, Miyagi prefecture	141° E, 38° N	2000	43	SND	Ando et al. (2016)
Bohai Sea	Panjin, Liaoning	122°8′ E, 40°39′ N	2008	20	PJ	This study
Yellow Sea	Dandong, Liaoning	124°22′ E, 40°7′ N	2008	18	DD	
	Yantai, Shandong	121°30′ E, 37°27′ N	2008	10	YT	
	Shidao, Shandong	120°11′ E, 35°45′ N	2008	12	SD	
East China Sea	Ningde, Fujian	119°40′ E, 26°38′ N	2008	18	ND	
	Fuqing, Fujian	119°38′ E, 25°34′ N	2007	13	FQ	

### 2.2 DNA amplification and sequencing

The mtDNA control region was amplified using the forward and reverse primers 5′‐GTT AGA GCG CCA GTC TTG TA‐3′ and 5′‐CCT GAA GTA GGA ACC AAA TGC‐3′, as described by Sekino et al. (2002). Amplification was performed in a 30 μl reaction volume containing 10 μM of each primer, 10 ng template DNA, 100 μM of each dNTP, 3 μl 10× PCR buffer containing MgCl_2_, and 1.25 U of DNA polymerase (Ex Taq™, Takara Co). The cycling conditions were as follows: preliminary denaturation at 94°C for 4 min, followed by strand denaturation at 94°C (45 s), annealing at 58°C (45 s), and primer extension at 72°C (1 min) for 33 cycles; and final elongation at 72°C (10 min). The PCR products were evaluated using 1% agarose gel electrophoresis. Products with a single bright band were selected and purified. The purified fragments were sequenced using an ABI‐3730 Automatic Sequencer (Applied Biosystems).

### 2.3 Data analysis

We pooled our sequences with an additional 233 sequences reported by Sekino et al. (2002) and Ando et al. (2016), corresponding to four locations (Table 1) on the coast of Japan (GenBank accession numbers: LC129561–LC129707). All sequences were screened, edited, and aligned in Lasergene 7.1 (DNAStar; Burland, 2000). A haplotype network was constructed in HapView, to examine genealogical relationships among haplotypes. All sequences were sorted into complete datasets based on their locations for population genetic analysis. Genetic diversity indices including the number of polymorphic sites (s), numbers of haplotypes (N), mean number of pairwise differences (k), nucleotide diversity (π), and haplotype diversity (h) were calculated for each population using Arlequin v3.5.2.2 (Excoffier & Lischer, 2010).

Genetic divergences between pairs of populations were tested by determining the pairwise fixation index, Φ_ST_, in Arlequin using the Kimura 2P substitution model; significance was tested by performing 10,000 permutations. When multiple comparisons were performed, *p* values were adjusted using the sequential Bonferroni procedure (Rice, 1989). Analysis of molecular variance was conducted to study hierarchical population structure and the geographic pattern of population subdivision (Excoffier et al., 1992). In this analysis, populations were grouped considering: (1) all populations, (2) populations in the marginal seas of the East China Sea, Yellow Sea, Bohai Sea, Northwest Pacific Ocean, and Sea of Japan. Meanwhile, spatial analysis of molecular variation (SAMOVA v2.0) was used to detect the geographic genetic structure. The number of groups ranged from 2 to 11, with 1000 permutations, and the maximum Φ_CT_ value (genetic diversity between groups) was calculated to determine the best way to group the population. A genetic distance matrix (Sunde et al., 2022) was created based on pairwise genetic divergences between populations. For geographic distance, the straight‐line distances between populations were calculated in terms of longitude and latitude. Mantel tests were used to detect isolation by distance (IBD) patterns in RStudio using the “geosphere” (Hijmans et al., 2017) and “vegan” package (Oksanen et al., 2013) using Spearman's method and 10,000 permutations. Geographic barriers were computed and visualized using Barrier v2.2 based on the Φst pairwise comparison matrix and geographic distance. This method applies the Monmonier's maximum distance algorithm to identify barriers to gene flow among sites, namely the zones in which differences between pairs of sites are the largest.

Phylogenetic analysis was conducted for haplotypes using the maximum likelihood (ML) method and Bayesian analyses (BI). The program ModelFinder (Kalyaanamoorthy et al., 2017) was used to determine the most appropriate model for the analyses using the Akaike Information Criterion (Sakamoto et al., 1986). As the optimum model of substitution, GTR + I + G4 + F (ML) and GTR + I + G + F (BI) were selected for the mtDNA control region. ML analyses were performed using IQ‐TREE v.1.6.8 (Nguyen et al., 2015) with 5000 bootstrap replicates to estimate node reliability. BI was performed using MrBayes v3.2.6 (Huelsenbeck & Ronquist, 2001). Four Monte Carlo Markov chains (MCMC) were run simultaneously with 1 × 10^8^ generations using default settings. As the outgroup, we used *Paralichthys lethostigma* (Jordan & Gilbert, 1884; Genbank accession number: DQ450964).

The number of genetically homogeneous groups (K) was identified by a model‐based clustering approach in the Structure 2.3.4 (Pritchard et al., 2000). In this approach, a model that allows the admixture LOCPRIOR option (Porras‐Hurtado et al., 2013) was used. Without any a priori information, replicates were run 10 times for a number of clusters, K, from 1 to 11, using 1,200,000 MCMC generations after an initial burn‐in of 200,000 replicates. The most likely value of K was determined in Structure Harvester Web 0.6.94 (Earl & VonHoldt, 2012) based on the ΔK method (Evanno et al., 2005) and the K with the highest likelihood (Pritchard et al., 2000). A structure distribution plot was drawn using Distruct 1.1 (Rosenberg, 2004).

A neutrality test (Excoffier & Lischer, 2010), mismatch distribution analysis (Rogers & Harpending, 1992), and Bayesian skyline plot (Ho & Shapiro, 2011) analysis were used to analyze the demographic history of *P. olivaceus*. The Tajima's *D* and Fu's *Fs* values were calculated to evaluate neutrality using Arlequin (Excoffier & Lischer, 2010), and the significance of the obtained values was tested by generating 1000 random samples under the null hypothesis of selective neutrality. The mismatch distribution analysis was carried out for the inference of historical population dynamics. Bayesian skyline plots were generated using BEAST v2.6.6 (Drummond et al., 2012) based on the GTR + I + G + F model using a strict molecular clock; the mutation rate of the control region was considered as 3.6%/Myr (Donaldson & Wilson Jr, 1999), and 5 × 10^8^ generations for MCMC were performed. The skyline plot was generated using Tracer v1.7.2 (Rambaut et al., 2018).

## 3 RESULTS

### 3.1 Genetic diversity

All sequences were aligned, and a 379‐bp segment of the mtDNA control region was obtained for 324 specimens. A total of 223 haplotypes were defined, and 251 conserved sites, 128 variable sites, and 92 parsimony informative sites were detected (Table 2). Most haplotypes (186) were found only in one population, among which 174 were singletons. Five were shared among four populations, four were shared among three populations, and 28 were shared among two populations. Haplotype diversity (h) ranged from 0.850 ± 0.078 (Ningde [ND]) to 0.998 ± 0.004 (Hokkaido). Nucleotide diversity (π) ranged from 0.017 ± 0.010 (Fuqing [FQ]) to 0.036 ± 0.020 (Yantai [YT]). Haplotype diversity was higher in the Japanese group than in the Chinese group. The number of polymorphic sites (s) and the number of haplotypes (N) showed the same trend. The mean number of pairwise differences (k) and nucleotide diversity (π) was lowest in the population samples from the East China Sea (FQ and ND, k = 6.462–10.052, π = 0.017–0.027). High nucleotide diversity and haplotype diversity were detected in each population (Table 2).

**TABLE 2:** Sampling sites, collection dates, sample size, genetic diversity indices, Tajima's *D*, Fu's *Fs*, and corresponding *p*‐value for each population of *Paralichthys olivaceus*.

Codes	No. of haplotypes (N)	No. of polymorphic sites (s)	Haplotype diversity (h)	Nucleotide diversity (π)	Mean number of pairwise differences (k)	Tajima's D	*p*‐value	Fu's Fs	*p*‐value
HKD	48	72	0.998 ± 0.004	0.029 ± 0.015	10.896 ± 5.039	−1.192	.085	−24.54	.000
TTR	64	79	0.998 ± 0.003	0.032 ± 0.016	12.284 ± 5.616	−0.909	.17	−24.372	.000
CHB	65	95	0.998 ± 0.003	0.032 ± 0.017	12.107 ± 5.537	−1.413	.049	−24.376	.000
SND	37	70	0.992 ± 0.007	0.031 ± 0.016	11.728 ± 5.415	−1.067	.141	−21.398	.000
PJ	8	33	0.879 ± 0.040	0.028 ± 0.015	10.647 ± 5.062	0.480	.759	3.789	.929
DD	15	46	0.980 ± 0.024	0.031 ± 0.017	11.935 ± 5.665	−0.460	.351	−3.120	.095
YT	9	41	0.978 ± 0.054	0.036 ± 0.020	13.756 ± 6.757	−0.196	.472	−0.915	.255
SD	10	38	0.970 ± 0.044	0.034 ± 0.019	13.182 ± 6.386	−0.099	.502	−0.747	.300
ND	10	31	0.850 ± 0.078	0.027 ± 0.014	10.052 ± 4.821	0.314	.715	0.966	.671
FQ	8	20	0.910 ± 0.056	0.017 ± 0.010	6.462 ± 3.271	0.011	.524	−0.024	.494
Total	223	130	0.996 ± 0.001	0.032 ± 0.016	12.029 ± 5.453	−1.326	.050	−23.831	.002

Abbreviations: CHB, Chiba Prefecture; DD, Dandong, Liaoning; FQ, Fuqing, Fujian; HKD, Hokkaido Prefecture; ND, Ningde, Fujian; PJ, Panjin, Liaoning; SD, Shidao, Shandong; SND, Sendai Bay, Miyagi prefecture; TTR, Tottori Prefecture; YT, Yantai, Shandong.

### 3.2 Population genetic structure

**FIGURE 2:**
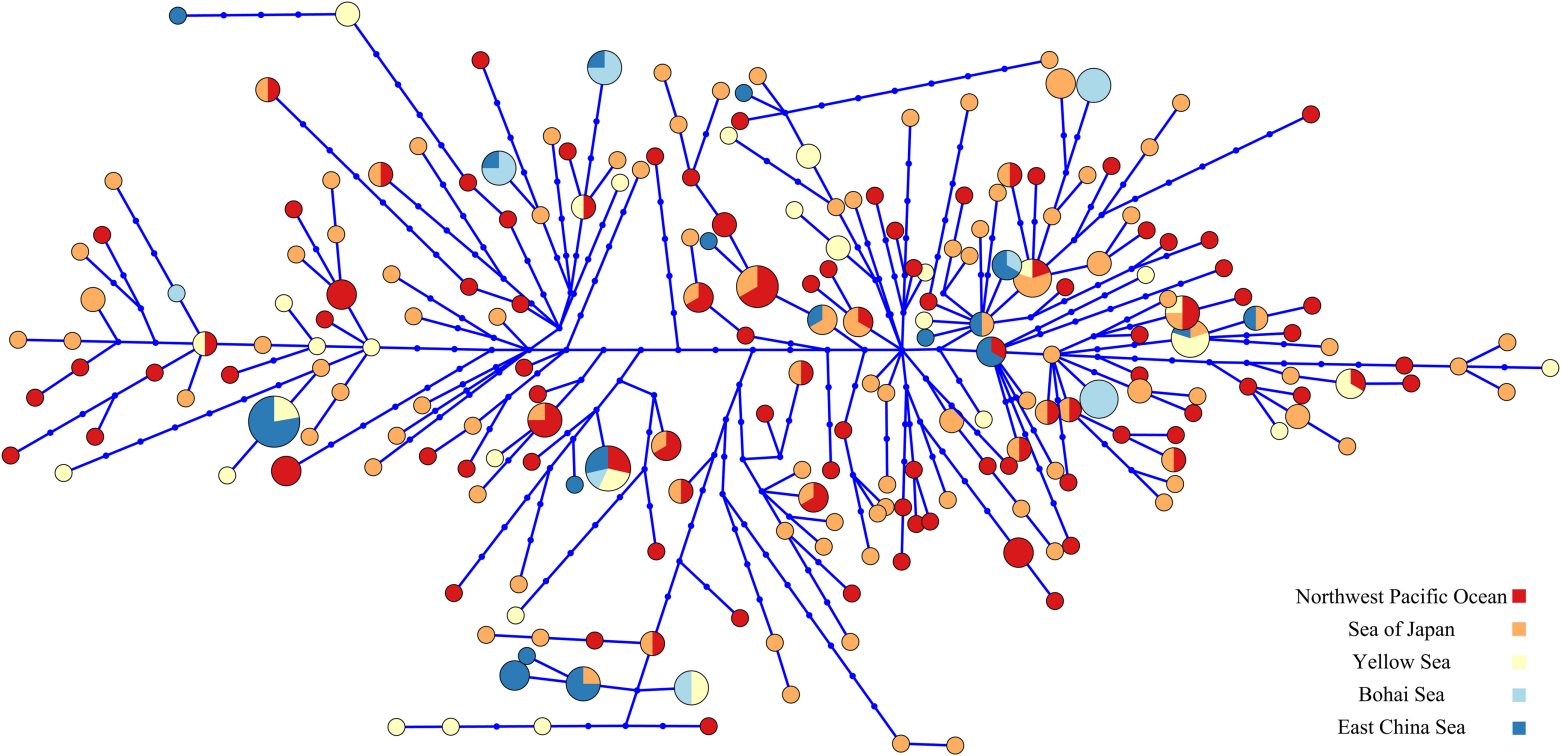
Phylogenetic relationships of *Paralichthys olivaceus* haplotypes represented in median‐joining haplotype network based on mitochondrial region D‐loop sequences. Each circle represents a unique haplotype, and the size of the circle is proportional to its total frequency. Each branch connecting different circles represents a single nucleotide change, and blue dot on the branches represent an additional nucleotide change. Colors denote sample geographic origins as indicated by the legend.

Phylogenetic trees of the haplotypes based on ML and BI analyses revealed no clusters corresponding to sampling locations or significant genealogical branches (Figures S1 and S2). The results from the structure assignment test supported three clusters (K = 3 as the highest value), but the peak of the delta k value was low. No distinct population genetic structure could be obviously distinguished by this assignment (Figure S3). The haplotype network showed that each geographical group had a mixed distribution pattern with no central haplotype, and the evolutionary relationship showed no genealogical branches (Figure 2; Table S1).

### 3.3 Population genetic differentiation

Among the 45 pairwise comparisons, 12 were significant after sequential Bonferroni correction. Pairwise Φ_ST_ values between the 10 populations ranged from −0.033 to 0.444 (Table 3). The largest difference was seen between the FQ and ND populations (Φ_ST_ = 0.444, *p* = .000). The 24 pairwise comparisons between the Japanese and Chinese populations revealed significant differences for eight comparisons, and Φ_ST_ values ranged from −0.022 to 0.221. FQ significantly differed from the other populations in pairwise comparison except for the YT. Most pairwise Φ_ST_ values were markedly low, and some were negative.

**TABLE 3:** Pairwise Φ_ST_ estimation (below diagonal) and its associated probability (above diagonal) among ten populations of *Paralichthys olivaceus* based on mtDNA control region data.

	HKD	TTR	CHB	SND	PJ	DD	YT	SD	ND	FQ
HKD		0.056	0.080	0.115	0.029	0.161	0.329	0.001***	0.000***	0.000***
TTR	0.013		0.434	0.604	0.239	0.366	0.850	0.018	0.001***	0.000***
CHB	0.010	−0.001		0.861	0.070	0.262	0.602	0.025	0.001***	0.000***
SND	0.011	−0.004	−0.007		0.157	0.249	0.779	0.036	0.003	0.000***
PJ	0.041	0.006	0.022	0.015		0.146	0.737	0.049	0.015	0.001***
DD	0.013	0.000	0.005	0.007	0.025		0.564	0.032	0.003	0.001***
YT	0.004	−0.022	−0.010	−0.021	−0.030	−0.013		0.205	0.058	0.004
SD	0.140	0.068	0.058	0.058	0.083	0.090	0.032		0.778	0.000***
ND	0.184	0.103	0.097	0.101	0.121	0.151	0.084	−0.033		0.000***
FQ	0.143	0.185	0.194	0.201	0.230	0.179	0.184	0.366	0.444	

Abbreviations: CHB, Chiba Prefecture; DD, Dandong, Liaoning; FQ, Fuqing, Fujian; HKD, Hokkaido Prefecture; ND, Ningde, Fujian; PJ, Panjin, Liaoning; SD, Shidao, Shandong; SND, Sendai Bay, Miyagi prefecture; TTR, Tottori Prefecture; YT, Yantai, Shandong. *Note*: Asterisks represent significance levels after sequential Bonferroni correction: ***p‐values ≤ .001.

**FIGURE 3:**
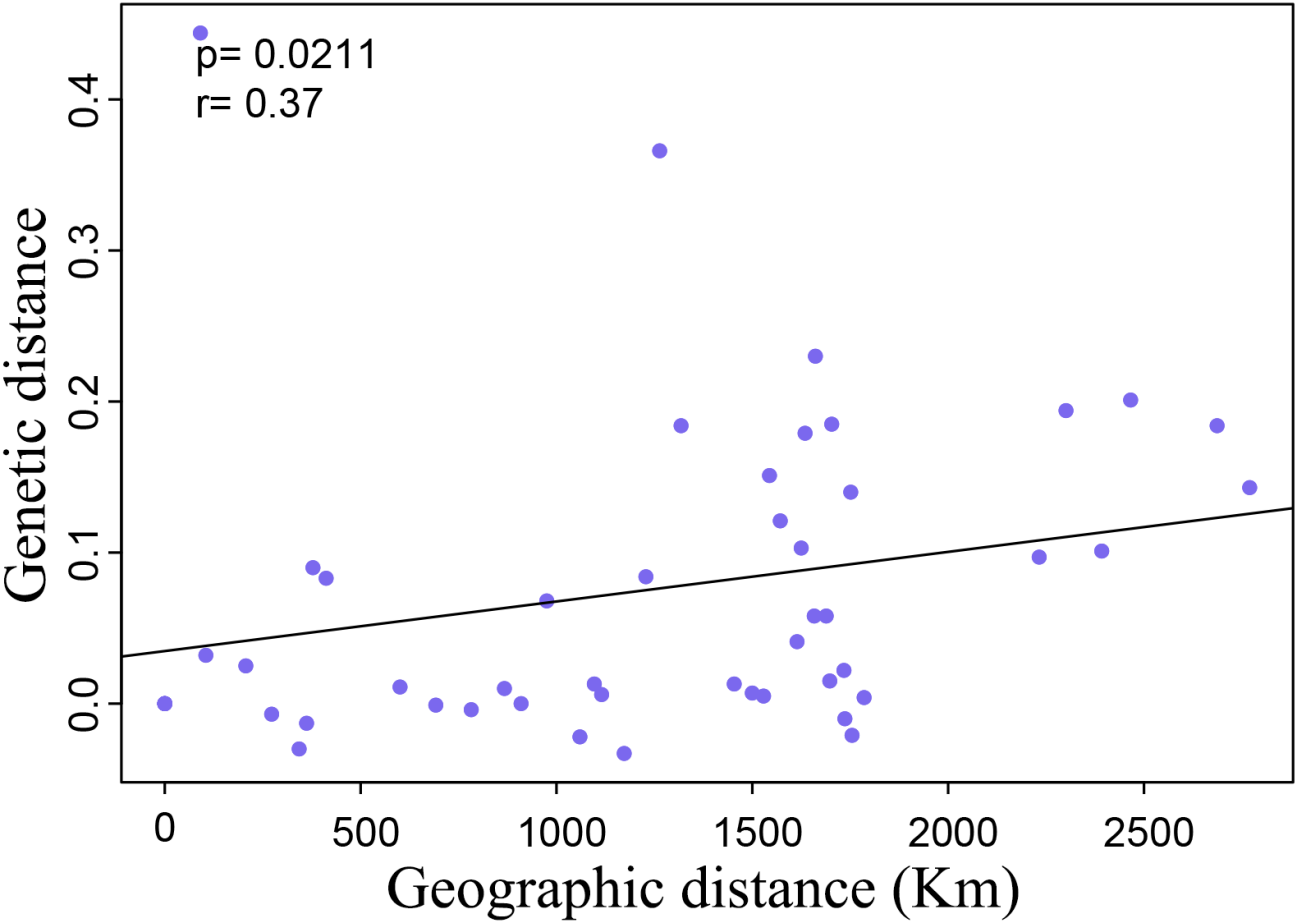
Isolation by distance analyses in *Paralichthys olivaceus* for all sites.

**FIGURE 4:**
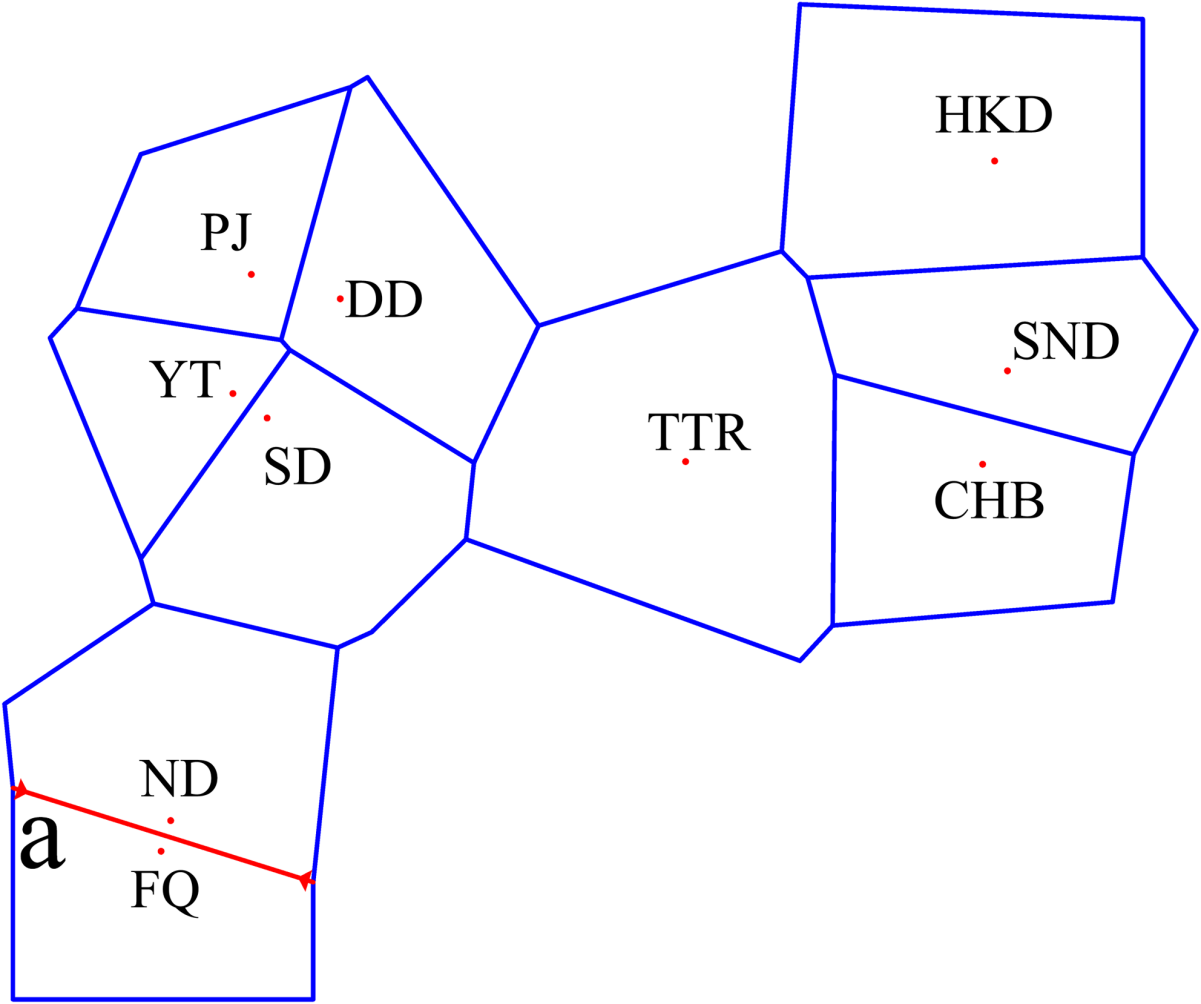
Results of the Barrier analysis showing the spatial separation of Paralichthys olivaceus populations. The populations are indicated by red points and uppercase letters. Lowercase letters indicate the order of the found barriers. Barriers are delimited by red lines. Blue lines indicate the Voronoi tessellation. Abbreviations: CHB, Chiba Prefecture; DD, Dandong, Liaoning; FQ, Fuqing, Fujian; HKD, Hokkaido Prefecture; ND, Ningde, Fujian; PJ, Panjin, Liaoning; SD, Shidao, Shandong; SND, Sendai Bay, Miyagi prefecture; TTR, Tottori Prefecture; YT, Yantai, Shandong.

An examination of the IBD patterns with Φ_ST_ revealed genetic differentiation that increases linearly as a function of geographic distance, as well as significant differences in population differentiation across sampling sites (*r* = 0.37, *p* = .021, Figure 3). According to the results of the barrier analysis, the priority barrier was observed between FQ and other populations, implying that genetic differentiation changes abruptly, and these abrupt changes are associated with barriers (Figure 4). Analysis of molecular variance revealed significant genetic differentiation among the nine populations (Φ_ST_ = 0.045, *p* < .001), among populations within five marginal seas (Φ_SC_ = 0.065, *p* < .001; Table 4). The best partitioning of the genetic structure was obtained when samples were divided into two groups, based on the largest value of Φ_CT_ (0.169). The first and second groups were composed of fish from the FQ and other populations, respectively. Results of the SAMOVA supported regional genetic subdivision as revealed by the geographic barrier analysis (Table 4).

**TABLE 4:** Analysis of molecular variance (AMOVA) and spatial analysis of molecular variance (SAMOVA) of *Paralichthys olivaceus* populations based on mtDNA control region data.

Analysis	Source of variance	df	Fixation index	*p*‐value	% variance
All sites	Among populations	9	Φ_ST_ = 0.045	.00	4.48
	Within populations	314			95.52
Marginal Sea groups	Among groups	4	Φ_CT_ = −0.026	.567	−2.56
	Within groups	5	Φ_SC_ = 0.065	.00	6.65
	Within populations	314	Φ_ST_ = 0.041	.00	95.91
SAMOVA two groups	Among groups	1	Φ_CT_ = 0.169	.098	16.88
(FQ and other populations)	Within groups	8	Φ_SC_ = 0.027	.00	2.27
	Within populations	314	Φ_ST_ = 0.192	.00	80.85

### 3.4 Demographic history

**FIGURE 5:**
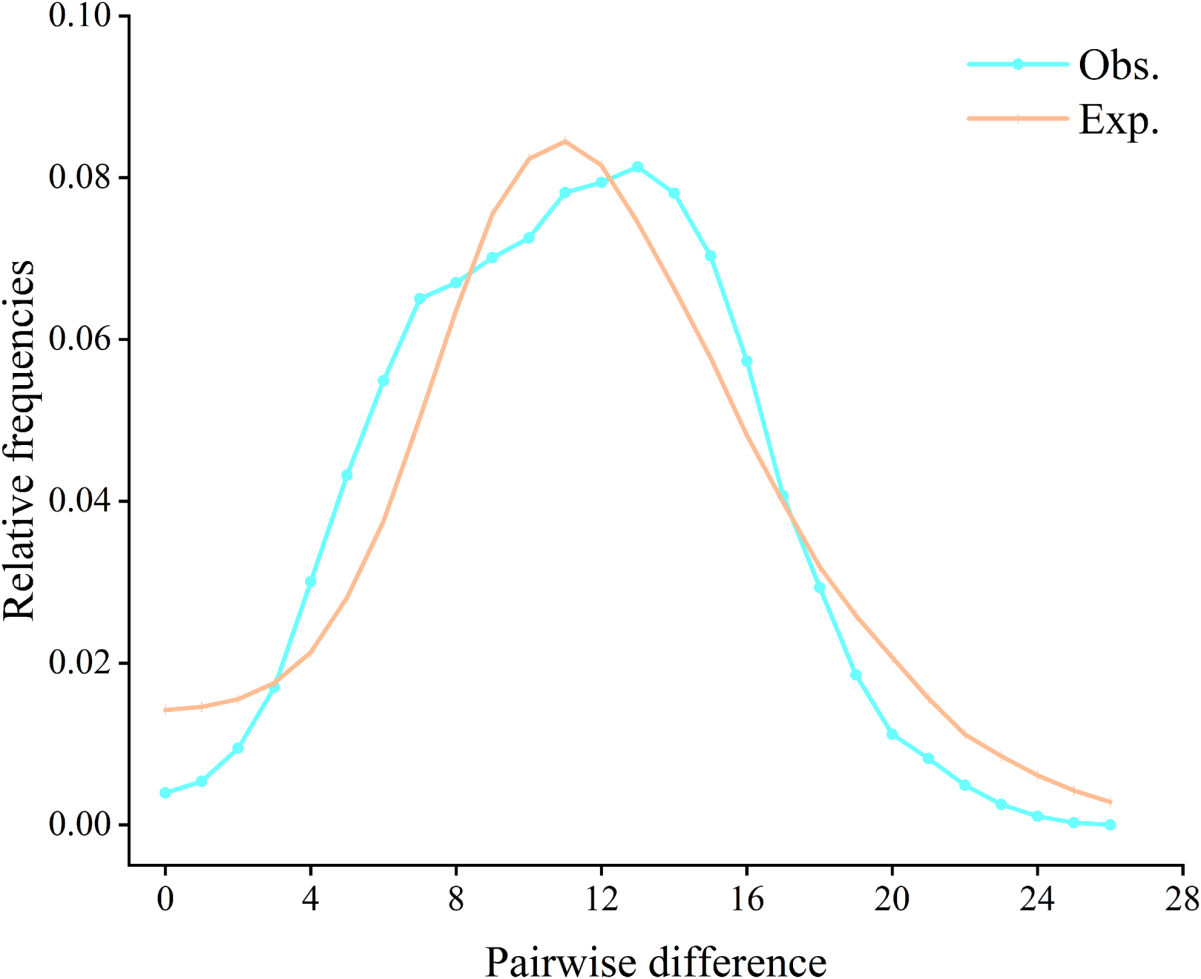
Mismatch distribution analysis determining a demographic expansion based on mtDNA control region.

**FIGURE 6:**
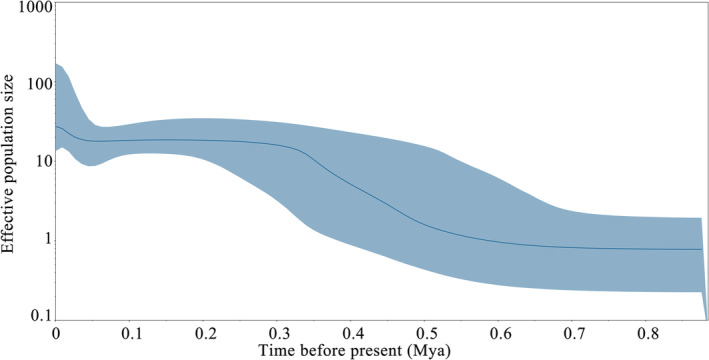
Bayesian skyline plots showing the effective female *Paralichthys olivaceus* population size through time based on the mtDNA control region. The solid line indicates the median estimates of NeT (Ne = effective female population size; T = generation time); blue shading represents the 95% confidence interval of NeT.

A unimodal distribution was observed in the mismatch distribution analysis (Figure 5). The neutrality test statistics (Tajima's *D* and Fu's *Fs*) were negative, and Fu's *Fs* significantly deviated from the neutral expectation (Tajima's *D* = −1.326, *p* = .050; Fu's *Fs* = −23.851, *p* = .002; Table 2). The results of the mismatch distribution analysis and neutrality test results fit the model of demographic growth rather than the model of constant population size. Bayesian skyline plots for all samples revealed demographic expansion in the middle and late Pleistocene (approximately 310,000–610,000 years ago) (Figure 6).

## 4 DISCUSSION

### 4.1 Genetic diversity

The mtDNA control regions of *P. olivaceus* in the Northwest Pacific Ocean showed high haplotype diversity (0.996 ± 0.001) and high nucleotide diversity (0.032 ± 0.016). The higher diversity may be that a large stable population with a long evolutionary history or secondary contacts among differentiated lineages (Grant & Bowen, 1998; Srinivas et al., 2021; Zhong et al., 2020). When combined with the results of Liu et al., 2019 and other analysis results of this study, it is shown that *P. olivaceus* has a large stable population and carries out panmixia within the distribution range. Li and Wang (1995) found that Chinese *P. olivaceus* was mainly distributed and spawned in the Yellow Sea and the Bohai Sea; this may be one of the reasons for the lower genetic diversity in the East China Sea. In addition, the two geographical populations in the East China Sea are in relatively closed bays and have no overwintering migration, potentially explaining their lower genetic diversity. The two geographical populations in the East China Sea are peripheral populations of this species range and have a low density, which may also contribute to their lower genetic diversity (García‐Ramos & Kirkpatrick, 1997). Artificial enhancement of *P. olivaceus* is performed more in the north than in the south of China, and little is performed in the East China Sea (Yang et al., 2013), indicating the lower diversity in the East China Sea is attributable to natural causes. This may also be caused by the stepping stone model or peripheral population. In the Last Glacial Maximum, the Bohai Sea became a wasteland (Wang, 1990), which inevitably led to the extinction of *P. olivaceus* in this region. After the last glacial period, *P. olivaceus* entered the Bohai Bay through Holocene Sea immersion, possibly leading to the lower genetic diversity of the Panjin population at the northeast end of this Bay. We speculate that the low diversity of the two populations in the East China Sea may also be caused by this reason.

### 4.2 Genetic structure and genetic differentiation

In the phylogenetic analysis, the 223 haplotypes were not clustered by location or marginal sea. The haplotype network and structure assignment test also showed no obvious structure or central haplotype. Thus, *P. olivaceus* does not have an obvious geographical structure. The dispersal of larvae with ocean currents is an important cause of the limited genetic differentiation of marine fishes that have a geographically large distribution range (Strathmann et al., 2002). It is generally thought that the levels of genetic differentiation among marine fish populations are low. Previous studies have reported that extensive gene exchange occurs over a wide geographical range in marine fishes (De Queiroz‐Brito et al., 2022; Grant & Bowen, 1998; Han et al., 2008; Song et al., 2010). The Φ_ST_ between the ND and FQ populations (East China Sea) was the highest value among all populations (Table 3), indicating the greatest genetic differentiation is between these populations. This result may be explained by the fact that the ND and FQ population samples were collected from completely closed waters, whereas the other population samples were collected from open waters. Combined with IBD, SAMOVA, and geographic barrier analysis, the results show that the FQ population has formed significant geographical isolation and genetic differentiation from other populations.

### 4.3 Demographic history

The population expansion of *P. olivaceus* was analyzed using three methods, haplotype nucleotide mismatch analysis, neutrality tests, and Bayesian skyline plots. Tajima's *D* neutrality test focuses on the ancient mutations in a population and can reflect population events over a long‐time scale. Fu's *Fs* neutrality test is sensitive to recent events affecting a population. Therefore, when the population accumulates large amounts of variation in a short time, Fu's *Fs* tends to give a large negative value (Su et al., 2001). Combined with haplotype nucleotide mismatch analysis, the results showed a unimodal distribution, indicating that the population recently experienced population expansion events. However, this is different from a demographic scenario assessed by genetic diversity. We hypothesized that it may be because *P. olivaceus* has a large enough population, and that the population expansion occurs in a small range, which has a small impact on the distribution range of the whole species.

The calculated population expansion time is approximately 310,000–610,000 years ago in the middle and late Pleistocene. Climatic fluctuations during the Pleistocene led to contraction and expansion of the distribution range of many species, which typically impact genetic diversity (Avise, 2000; Hewitt, 2000; Li, 1997). In the Last Glacial Maximum, the Yellow Sea and the Bohai Sea became land, and the East China Sea was approximately 50% of its current size (Wang, 1990). The survival range of marine fish decreased sharply; therefore, the *P. olivaceus* population may have been isolated in one or more glacial refugia, and the Northwest Pacific Ocean population may have experienced bottleneck effects.

The marine environment is a very open setting. In marine fish, gene exchange between populations is likely affected by various marine environment‐related factors, including geographical distance, ocean circulation, seawater temperature, and salinity (Han et al., 2012). Historical migration trends may be related to major sea‐level cycles that occur at intervals of ∼100 kyr over the past ∼800 kyr, with maximum amplitudes of 120–140 m (Imbrie et al., 1992; Lambeck et al., 2002). Many studies have shown a weak genetic differentiation between the geographical populations of surface marine fish that can migrate long distances or swim. This is because of the free diffusion of floating eggs, fish larvae, and juveniles or adults, and few geographical or other obstacles in the open ocean environment, enabling widespread and extensive gene exchange in these marine fish (Canfield et al., 2022; Hewitt, 2000; Palumbi, 1994; Pérez‐Rodríguez et al., 2021). However, *P. olivaceus* is a benthic fish, and its life history indicates no long distance migration habit. Therefore, the reason for panmixia among populations may be related to their early life habits. Active diffusion of fish larvae and juveniles as well as marine environmental factors, such as ocean circulation and climate change in the late Pleistocene, played important roles in shaping the systematic geographical pattern and population genetic structure of *P. olivaceus*.

### 4.4 Genetic resource conservation

Based on our results, the FQ population exhibits the lowest genetic diversity and highest differentiation and should be prioritized for protection to avoid further loss of genetic diversity. The mtDNA control region may exhibit hypervariability and homoplasy, which cannot accurately reflect the overall population structure (Takeshima et al., 2005; Verma et al., 2016). To better manage, protect, and utilize *P. olivaceus*, larger numbers of polymorphic loci from the nuclear genome combined with multiple mtDNA (COI, ND2, ND5) regions should be collected to investigate its population genetics. These loci can be used to assess contemporary population connectivity and genetic differentiation. By combining traditional fishery investigations, physiological and ecological information, and molecular biology methods, the resource status of *P. olivaceus* can be determined. This study not only has basic theoretical significance for understanding the historical evolution of marine fish populations in the Northwest Pacific but also reveals how fish respond to climate and environmental changes, which is of great practical value for the rational development and formulation of effective species protection strategies.

## AUTHOR CONTRIBUTIONS

**Cheng‐He Sun:** Formal analysis (lead); methodology (equal); validation (lead); visualization (lead); writing – original draft (lead); writing – review and editing (lead). **Fan Yang:** Investigation (supporting); writing – review and editing (supporting). **Qi Huang:** Formal analysis (supporting); writing – review and editing (supporting). **Xiao‐Shu Zeng:** Investigation (supporting); writing – review and editing (supporting). **Ya‐Nan Zhang:** Visualization (supporting); writing – review and editing (supporting). **Sha Li:** Funding acquisition (supporting); writing – review and editing (supporting). **Jian‐Feng Yu:** Visualization (supporting); writing – review and editing (supporting). **Qun Zhang:** Conceptualization (lead); data curation (lead); funding acquisition (lead); investigation (lead); project administration (lead); writing – review and editing (supporting).

## CONFLICT OF INTEREST

The authors declare that they have no competing interests.

## Supporting information


**Table S1.** Distribution of *Paralichthys olivaceus* haplotypes among different localities.
**Figure S1**. Maximum likelihood trees constructed based on mtDNA control region haplotypes.
**Figure S2**. Bayesian interference trees constructed based on mtDNA control region haplotypes.
**Figure S3**. Results of Bayesian clustering analysis of mtDNA control regions in *Paralichthys olivaceus* populations (conducted using STRUCTURE). The changes in ΔK (A) and lnP(K) (B) in different clustering situations, K. The ΔK plot shows that the highest ΔK value occurs at K = 3. This plot also shows lnP(K), which demonstrates the increase in the posterior probability of K. The clustering patterns of genetic components by three groups (K = 3) (including all the samples)(C).

## Data Availability

The data that support the findings of this study are openly available in: Researchgate https://doi.org/10.13140/RG.2.2.35864.67845, GenBank with accession numbers ON815658 ‐ ON815880 and, FigShare https://doi.org/10.6084/m9.figshare.19609341.
