# Supplementary material for: Genetic population structure and demographic history of the endemic fish Paralichthys olivaceus of the Northwest Pacific Ocean
Source: Ecol Evol. 2022 Nov 15;12(11):e9506. doi: 10.1002/ece3.9506 (PMC9666908; doi:10.1002/ece3.9506)
Supplement: Supplementary file 1 — Table S1. Distribution of Paralichthys olivaceus haplotypes among different localities. Figure S1. Maximum likelihood trees constructed based on mtDNA control region haplotypes. Figure S2. Bayesian interference trees constructed based on mtDNA control region haplotypes. Figure S3. Results of Bayesian clustering analysis of mtDNA control regions in Paralichthys olivaceus populations (conducted using STRUCTURE). The changes in ΔK (A) and lnP(K) (B) in different clustering situations, K. The ΔK plot shows that the highest ΔK value occurs at K = 3. This plot also shows lnP(K), which demonstrates the increase in the posterior probability of K. The clustering patterns of genetic components by three groups (K = 3) (including all the samples)(C). [file ECE3-12-e9506-s001.docx]

Supplementary Material

**Supplementary Table 1.** Distribution of *Paralichthys olivaceus* haplotypes among different localities.

| CHB | Count |  | HKD | Count |  | TTR | Count |  | SND | Count |  | DD | Count |
| --- | --- | --- | --- | --- | --- | --- | --- | --- | --- | --- | --- | --- | --- |
| Hap_1 | 1 |  | Hap_13 | 1 |  | Hap_18 | 1 |  | Hap_12 | 2 |  | Hap_28 | 2 |
| Hap_2 | 1 |  | Hap_20 | 1 |  | Hap_20 | 1 |  | Hap_20 | 2 |  | Hap_98 | 1 |
| Hap_3 | 1 |  | Hap_30 | 1 |  | Hap_31 | 1 |  | Hap_25 | 2 |  | Hap_187 | 1 |
| Hap_4 | 1 |  | Hap_45 | 1 |  | Hap_36 | 1 |  | Hap_30 | 2 |  | Hap_188 | 2 |
| Hap_5 | 1 |  | Hap_62 | 1 |  | Hap_37 | 1 |  | Hap_65 | 1 |  | Hap_189 | 1 |
| Hap_6 | 1 |  | Hap_66 | 1 |  | Hap_42 | 1 |  | Hap_93 | 1 |  | Hap_190 | 1 |
| Hap_7 | 2 |  | Hap_67 | 1 |  | Hap_59 | 1 |  | Hap_98 | 1 |  | Hap_191 | 1 |
| Hap_8 | 1 |  | Hap_68 | 1 |  | Hap_62 | 1 |  | Hap_106 | 1 |  | Hap_192 | 1 |
| Hap_9 | 1 |  | Hap_69 | 1 |  | Hap_65 | 1 |  | Hap_118 | 1 |  | Hap_193 | 1 |
| Hap_10 | 1 |  | Hap_70 | 1 |  | Hap_91 | 1 |  | Hap_136 | 1 |  | Hap_194 | 1 |
| Hap_11 | 1 |  | Hap_71 | 2 |  | Hap_98 | 2 |  | Hap_147 | 1 |  | Hap_195 | 2 |
| Hap_12 | 1 |  | Hap_72 | 1 |  | Hap_99 | 2 |  | Hap_161 | 1 |  | Hap_196 | 1 |
| Hap_13 | 1 |  | Hap_73 | 1 |  | Hap_109 | 2 |  | Hap_162 | 1 |  | Hap_197 | 1 |
| Hap_14 | 1 |  | Hap_74 | 1 |  | Hap_110 | 1 |  | Hap_163 | 1 |  | Hap_198 | 1 |
| Hap_15 | 1 |  | Hap_75 | 1 |  | Hap_111 | 1 |  | Hap_164 | 1 |  | Hap_199 | 1 |
| Hap_16 | 1 |  | Hap_76 | 1 |  | Hap_112 | 1 |  | Hap_165 | 1 |  |  |  |
| Hap_17 | 1 |  | Hap_77 | 1 |  | Hap_113 | 1 |  | Hap_166 | 3 |  | YT | Count |
| Hap_18 | 2 |  | Hap_78 | 1 |  | Hap_114 | 1 |  | Hap_167 | 1 |  | Hap_65 | 1 |
| Hap_19 | 1 |  | Hap_79 | 1 |  | Hap_115 | 1 |  | Hap_168 | 1 |  | Hap_105 | 2 |
| Hap_20 | 2 |  | Hap_80 | 1 |  | Hap_116 | 1 |  | Hap_169 | 1 |  | Hap_178 | 1 |
| Hap_21 | 1 |  | Hap_81 | 1 |  | Hap_117 | 1 |  | Hap_170 | 1 |  | Hap_205 | 1 |
| Hap_22 | 1 |  | Hap_82 | 1 |  | Hap_118 | 1 |  | Hap_171 | 1 |  | Hap_207 | 1 |
| Hap_23 | 1 |  | Hap_83 | 1 |  | Hap_119 | 1 |  | Hap_172 | 1 |  | Hap_208 | 1 |
| Hap_24 | 1 |  | Hap_84 | 1 |  | Hap_120 | 1 |  | Hap_173 | 1 |  | Hap_209 | 1 |
| Hap_25 | 1 |  | Hap_85 | 1 |  | Hap_121 | 2 |  | Hap_174 | 1 |  | Hap_210 | 1 |
| Hap_26 | 1 |  | Hap_86 | 1 |  | Hap_122 | 1 |  | Hap_175 | 1 |  | Hap_211 | 1 |
| Hap_27 | 1 |  | Hap_87 | 1 |  | Hap_123 | 1 |  | Hap_176 | 1 |  |  |  |
| Hap_28 | 1 |  | Hap_88 | 1 |  | Hap_124 | 1 |  | Hap_177 | 1 |  | SD | Count |
| Hap_29 | 1 |  | Hap_89 | 1 |  | Hap_125 | 1 |  | Hap_178 | 1 |  | Hap_7 | 2 |
| Hap_30 | 1 |  | Hap_90 | 1 |  | Hap_126 | 1 |  | Hap_179 | 1 |  | Hap_105 | 1 |
| Hap_31 | 1 |  | Hap_91 | 1 |  | Hap_127 | 1 |  | Hap_180 | 1 |  | Hap_183 | 1 |
| Hap_32 | 1 |  | Hap_92 | 1 |  | Hap_128 | 1 |  | Hap_181 | 1 |  | Hap_205 | 1 |
| Hap_33 | 1 |  | Hap_93 | 1 |  | Hap_129 | 1 |  | Hap_182 | 1 |  | Hap_211 | 1 |
| Hap_34 | 1 |  | Hap_94 | 1 |  | Hap_130 | 1 |  | Hap_183 | 1 |  | Hap_212 | 2 |
| Hap_35 | 1 |  | Hap_95 | 1 |  | Hap_131 | 2 |  | Hap_184 | 1 |  | Hap_213 | 1 |
| Hap_36 | 1 |  | Hap_96 | 1 |  | Hap_132 | 1 |  | Hap_185 | 1 |  | Hap_214 | 1 |
| Hap_37 | 1 |  | Hap_97 | 1 |  | Hap_133 | 1 |  | Hap_186 | 1 |  | Hap_215 | 1 |
| Hap_38 | 1 |  | Hap_98 | 1 |  | Hap_134 | 1 |  |  |  |  | Hap_216 | 1 |
| Hap_39 | 1 |  | Hap_99 | 1 |  | Hap_135 | 1 |  |  |  |  |  |  |
| Hap_40 | 1 |  | Hap_100 | 1 |  | Hap_136 | 1 |  | PJ | Count |  | ND | Count |
| Hap_41 | 1 |  | Hap_101 | 1 |  | Hap_137 | 1 |  | Hap_7 | 1 |  | Hap_7 | 2 |
| Hap_42 | 2 |  | Hap_102 | 1 |  | Hap_138 | 1 |  | Hap_200 | 3 |  | Hap_82 | 1 |
| Hap_43 | 1 |  | Hap_103 | 2 |  | Hap_139 | 1 |  | Hap_201 | 5 |  | Hap_105 | 1 |
| Hap_44 | 1 |  | Hap_104 | 1 |  | Hap_140 | 1 |  | Hap_202 | 4 |  | Hap_160 | 1 |
| Hap_45 | 2 |  | Hap_105 | 1 |  | Hap_141 | 1 |  | Hap_203 | 1 |  | Hap_200 | 1 |
| Hap_46 | 1 |  | Hap_106 | 1 |  | Hap_142 | 1 |  | Hap_204 | 3 |  | Hap_203 | 2 |
| Hap_47 | 1 |  | Hap_107 | 1 |  | Hap_143 | 1 |  | Hap_205 | 2 |  | Hap_204 | 1 |
| Hap_48 | 2 |  | Hap_108 | 1 |  | Hap_144 | 1 |  | Hap_206 | 1 |  | Hap_211 | 7 |
| Hap_49 | 1 |  |  |  |  | Hap_145 | 1 |  |  |  |  | Hap_217 | 1 |
| Hap_50 | 1 |  |  |  |  | Hap_146 | 1 |  |  |  |  | Hap_218 | 1 |
| Hap_51 | 1 |  |  |  |  | Hap_147 | 1 |  |  |  |  |  |  |
| Hap_52 | 1 |  |  |  |  | Hap_148 | 1 |  |  |  |  | FQ | Count |
| Hap_53 | 1 |  |  |  |  | Hap_149 | 1 |  |  |  |  | Hap_66 | 3 |
| Hap_54 | 1 |  |  |  |  | Hap_150 | 1 |  |  |  |  | Hap_103 | 1 |
| Hap_55 | 1 |  |  |  |  | Hap_151 | 1 |  |  |  |  | Hap_175 | 2 |
| Hap_56 | 1 |  |  |  |  | Hap_152 | 1 |  |  |  |  | Hap_219 | 1 |
| Hap_57 | 1 |  |  |  |  | Hap_153 | 1 |  |  |  |  | Hap_220 | 3 |
| Hap_58 | 1 |  |  |  |  | Hap_154 | 1 |  |  |  |  | Hap_221 | 1 |
| Hap_59 | 1 |  |  |  |  | Hap_155 | 1 |  |  |  |  | Hap_222 | 1 |
| Hap_60 | 1 |  |  |  |  | Hap_156 | 1 |  |  |  |  | Hap_223 | 1 |
| Hap_61 | 1 |  |  |  |  | Hap_157 | 1 |  |  |  |  |  |  |
| Hap_62 | 1 |  |  |  |  | Hap_158 | 1 |  |  |  |  |  |  |
| Hap_63 | 1 |  |  |  |  | Hap_159 | 1 |  |  |  |  |  |  |
| Hap_64 | 1 |  |  |  |  | Hap_160 | 1 |  |  |  |  |  |  |
| Hap_65 | 1 |  |  |  |  |  |  |  |  |  |  |  |  |

Abbreviations: CHB, Chiba Prefecture; DD, Dandong, Liaoning; FQ, Fuqing, Fujian; HKD, Hokkaido Prefecture; ND, Ningde, Fujian; PJ, Panjin, Liaoning; SD, Shidao, Shandong; SND, Sendai Bay, Miyagi prefecture; TTR, Tottori Prefecture; YT, Yantai, Shandong.


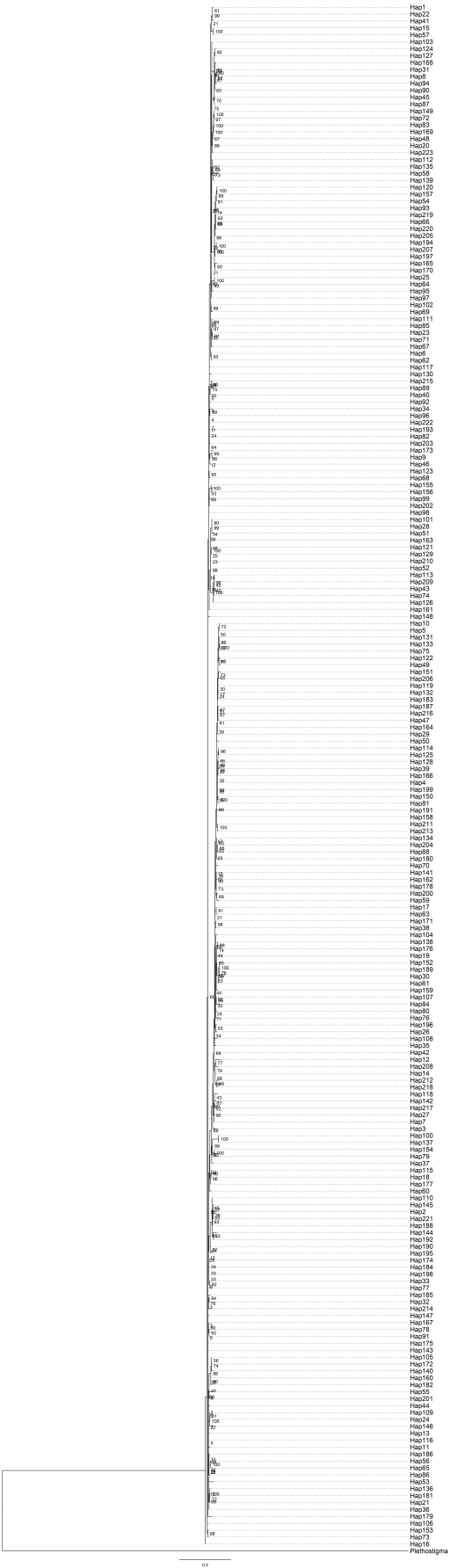


**Supplementary Figure 1**. Maximum likelihood trees constructed based on mtDNA control region haplotypes.


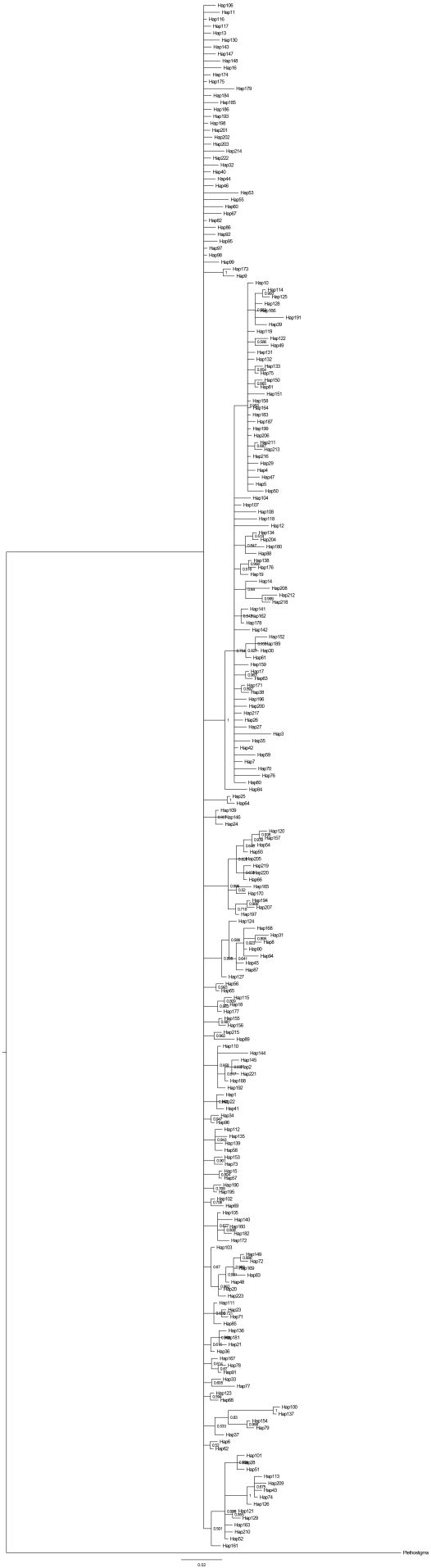


**Supplementary Figure 2**. Bayesian interference trees constructed based on mtDNA control region haplotypes.

**
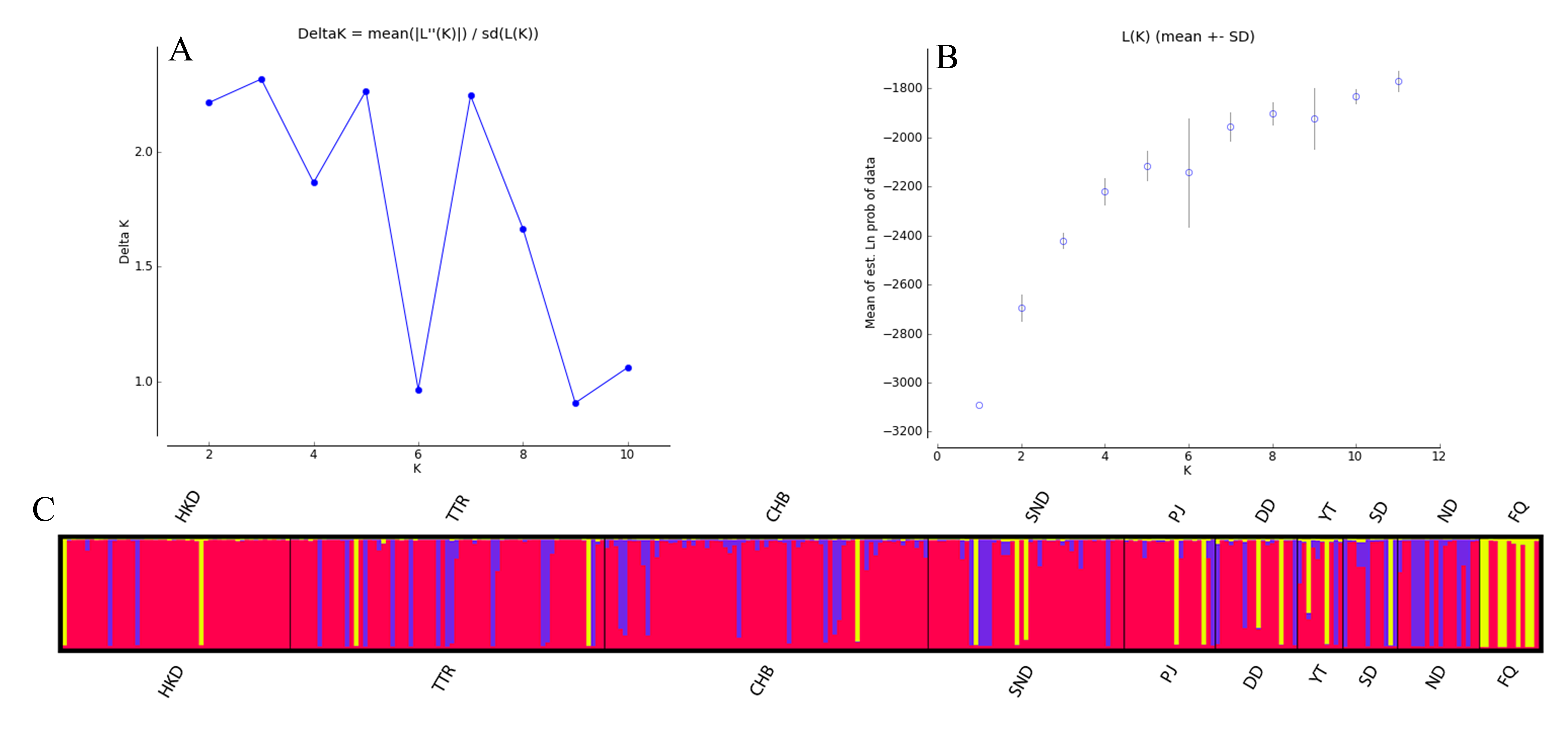
**

**Supplementary Figure 3.** Results of Bayesian clustering analysis of mtDNA control regions in *Paralichthys olivaceus* populations (conducted using STRUCTURE). The changes in ΔK (A) and lnP(K) (B) in different clustering situations, K. The ΔK plot shows that the highest ΔK value occurs at K = 3. This plot also shows lnP(K), which demonstrates the increase in the posterior probability of K. The clustering patterns of genetic components by three groups (K = 3) (including all the samples)(C).
